# Determinants of mortality among pediatric patients admitted to Wolaita Sodo University Comprehensive Specialized Hospital with acute bacterial meningitis, Southern Ethiopia: an unmatched case–control study

**DOI:** 10.1186/s12887-023-04410-6

**Published:** 2023-12-04

**Authors:** Ushula Deboch Borko, Temesgen Bati Gelgelu, Zewde Zema, Afework Alemu, Getahun Dendir, Eskinder Israel, Temesgen Lera Abiso, Beshada Zerfu Woldegeorgis

**Affiliations:** 1 School of Medicine, College of Health Science and Medicine, Wolaita Sodo University, Wolaita Sodo, Ethiopia; 2 School of Public Health, College of Health Science and Medicine, Wolaita Sodo University, Wolaita Sodo, Ethiopia; 3 School of Pharmacy, College of Health Science and Medicine, Wolaita Sodo University, Wolaita Sodo, Ethiopia; 4 School of Anesthesia, College of Health Science and Medicine, Wolaita Sodo University, Wolaita Sodo, Ethiopia

**Keywords:** Acute bacterial meningitis, Mortality, Pediatrics, Wolaita, Ethiopia

## Abstract

**Background:**

People of all ages suffer from acute bacterial meningitis, but children are the most vulnerable, accounting for over 50% of all cases and deaths in children under the age of five. It is the leading cause of morbidity, mortality, and long-term suffering worldwide. Children are at great risk of disease and mortality due to a lack of specific immunity associated with their young age. As a result, determinants of death were found among pediatric patients treated with acute bacterial meningitis at Wolaita Sodo University Comprehensive Specialized Hospital in Southern Ethiopia.

**Methods:**

A facility-based unmatched case–control study was conducted on pediatric patients admitted with acute bacterial meningitis at Wolaita Sodo University Comprehensive Specialized Hospital from July 1, 2019, to June 30, 2022. A total of 355 (71 cases and 284 controls) pediatric medical charts were used for data extraction using a preestablished checklist. Data were checked for completeness and consistency, entered into Epi-Data version 4.6 software, and transported to SPSS version 25 for analysis. Multivariable logistic regression analysis was performed to identify the independent determinants of acute bacterial meningitis mortality at a *P* value of < 0.05 along with a 95% confidence interval (CI).

**Results:**

Age between 2 months and 5 years (adjusted odds ratio (AOR) = 3.19, 95% CI = 1.15–8.88), admission in the summer season (AOR = 0.27, 95% CI = 0.15–0.49), and family size greater than or equal to six (AOR = 3.13, 95% CI = 1.76–5.56), initial antibiotic change (AOR = 10.81, 95% CI = 2.10–55.7), clinical features at presentation such as loss of consciousness (AOR = 16.90, 95% CI = 4.70–60.4), abnormal body movements (seizures) (AOR = 6.51, 95% CI = 1.82–23.4), increased intracranial pressure (AOR = 3.63, 95% CI = 1.78–7.4), malnutrition (AOR = 2.98, 95% CI = 1.34–6.59) and presence of more than one comorbidity (AOR = 3.03, 95% CI = 1.03–9.03) were found to be determinants of acute bacterial meningitis mortality.

**Conclusions:**

In summary, children aged 2 months to 5 years from large families ( > = 6) with a history of initial antibiotic change, malnutrition, more than one comorbidity, and worse clinical characteristics were related to greater death due to acute bacterial mortality in this study.

**Supplementary Information:**

The online version contains supplementary material available at 10.1186/s12887-023-04410-6.

## Introduction

Meningitis is an inflammatory disease of the leptomeninges (the tissues that cover the brain and spinal cord) [1]. Meningitis can be caused by infectious and noninfectious etiologies. Infectious causes include bacteria, viruses, fungi, or parasites, while autoimmunity, cancer, or reactions to medications are noninfectious agents [2, 3]. Of these, bacterial meningitis is the leading cause of death [4, 5]. If untreated, bacterial meningitis can be deadly in 50% of cases, and 10–20% of survivors are at risk of lasting sequelae such as brain damage, hearing loss, disability, and learning impairments [6]. Children are at great risk of disease and mortality due to a lack of specific immunity associated with their young age [6].

There were approximately 236,000 deaths with 2.5 million new cases from all forms of meningitis (excluding tuberculosis and cryptococcal meningitis) in 2019 [7, 8]. Furthermore, meningitis ranked sixth among the main causes of disability-adjusted life years among children under the age of 10 in 2019 [9]. The burden remains a source of concern in low- and middle-income countries [7, 10].

In Africa, from 1928 to 2018, approximately 2,628,283 meningitis cases, including 151,808 deaths, were reported to the World Health Organization African Region [11], with the highest burden of bacterial meningitis in an area of sub-Saharan Africa (SSA) extending from Ethiopia in the east to Gambia in the west, which is known as the “meningitis belt” because of its young population and its high prevalence of endemic diseases with regular epidemics caused by Neisseria meningitides [12–14].

The average fatality of meningitis in Africa from 1928 to 2018 was approximately 5.77% [11], with the highest burden in SSA known as the meningitis belt [15].

Ethiopia, an SSA country, has a high bacterial meningitis burden [16]. Approximately 6-8% of pediatric hospitalizations have case fatality rates ranging from 3.7 to 33.6%, placing Ethiopia.

among the top ten nations in the world with the greatest mortality rate from acute bacterial meningitis [17].

The literature has demonstrated that pediatric age groups, male sex, and countries with low and middle income were more affected than high-income countries [7, 18, 19]. The majority of deaths, approximately 70% of all meningitis/encephalitis deaths, occurred in children under five years of age in 12 countries, including Ethiopia [17, 20]. In addition, pediatric patients admitted during the summer season and whose residences were rural were associated with higher acute bacterial meningitis mortality [20, 21]. Bacterial meningitis death was high, with the number of siblings greater than 3 [22–24].

Furthermore, bacterial meningitis mortality among children with seizures, elevated intracranial pressure (ICP), and coma (loss of consciousness) ranged from 10.5–25% [16, 20, 25–28]. A delayed presentation period of more than 3–5 days before hospitalization was related to a significant fatality rate due to acute bacterial meningitis [24, 26, 29].

Malnourished children with acute bacterial meningitis had higher mortality (33.3%) [24, 26, 30].

Antibiotic regimen changes and bacterial species were associated with bacterial meningitis outcomes [22, 24, 31, 32].

To the best of our knowledge, limited studies have been performed in our country regarding the prevalence, etiology, diagnosis, and treatment outcomes of bacterial meningitis [16, 20, 21]. However, those studies did not agree on specific predictors associated with mortality due to acute bacterial meningitis among hospitalized pediatric patients. Therefore, the need for further study in our setup was unquestionable to determine factors associated with mortality among children aged 1 day to 14 years admitted with acute bacterial meningitis in Wolaita Sodo University Comprehensive Specialized Hospital (WSUCSH).

## Methods

### Study design

A facility-based unmatched case–control study was employed.

### Study setting

This study was conducted at WSUCSH from July 01, 2019, to June 30, 2022. The WSUCSH is located in Soho town, the capital of the Wolaita Zone, approximately 320 km south of Ethiopia’s capital, Addis Ababa. Currently, the hospital has 370 beds with 975 health professionals and 760 nonhealth (supportive) staff serving approximately more than 5 million people in Wolaita and neighboring zones of the catchment population.

### Participants

All pediatric patients hospitalized with acute bacterial meningitis in WSUCSH from July 1, 2019, to June 30, 2022, were considered as the source population. while.

The study population included all pediatric patients hospitalized with a presumptive (physician’s clinical or laboratory-based) diagnosis of acute bacterial meningitis between July 1, 2019, and June 30, 2022. Moreover,

Pediatric deaths due to acute bacterial meningitis confirmed by a physician from July 1, 2019, to June 30, 2022, were considered cases, while those who recovered or were alive at discharge and met the inclusion criteria were considered controls.

### Inclusion and exclusion criteria

The study included pediatric patients aged between 1 day and 14 years who were probable or confirmed to have acute bacterial meningitis and whose charts contained comprehensive medical information about issues relating to diagnosis, treatment, and discharge outcome (dead or alive).

Recorded data of pediatrics with no clear end outcome or incomplete chart or referrals or those who were discharged against medical advice after ABM diagnosis, whose antibiotic treatment was discontinued together with confirmed alternative diagnosis as well as a diagnosis of tuberculosis meningitis based on positive acid fast bacilli (AFB) from CSF gene x-pert test results were excluded.

### Sample size determination and sampling procedure

**Fig. 1:**
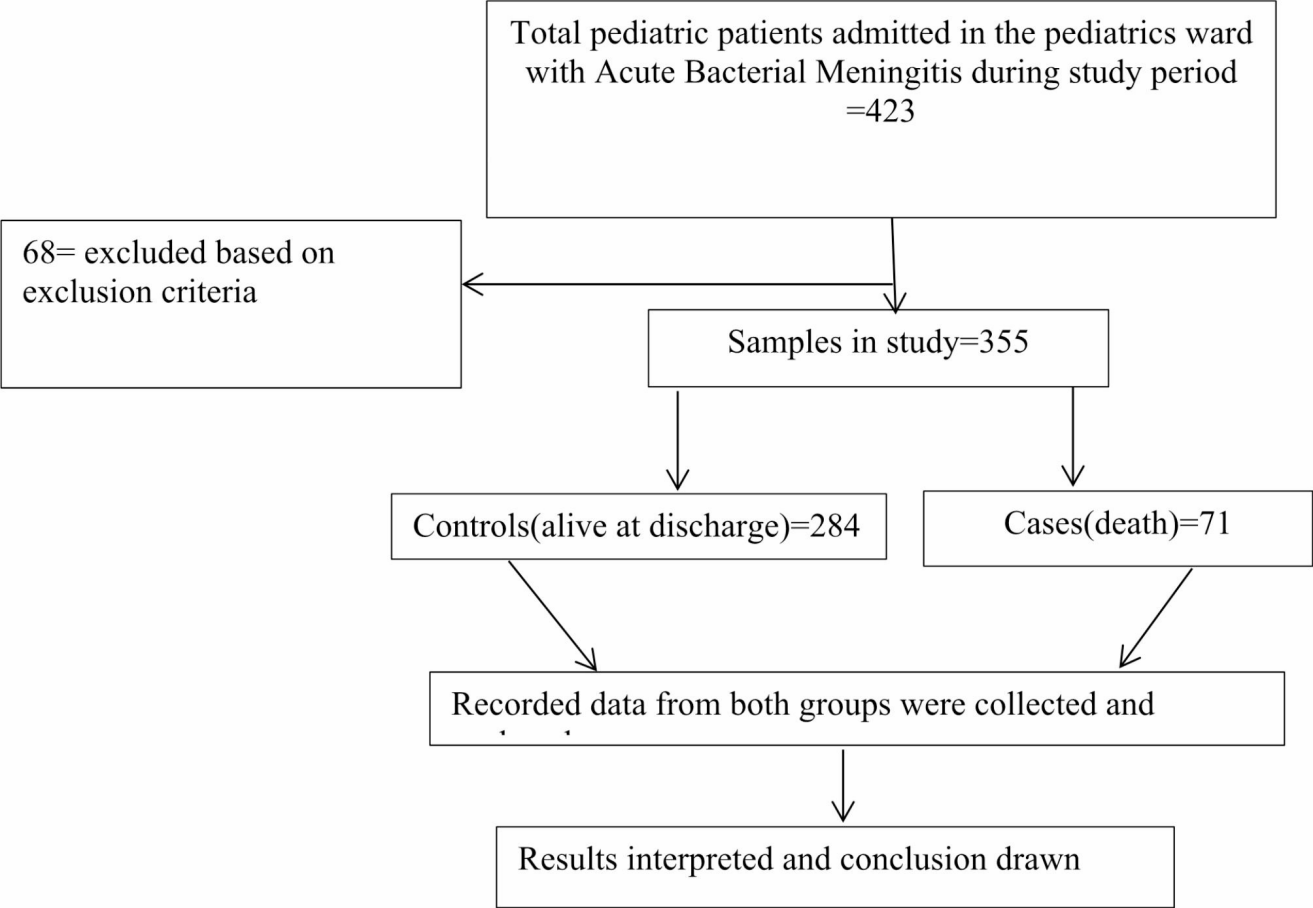
Flow diagram depicting charts enrolled in a study with acute bacterial meningitis at Wolaita Sodo University Comprehensive Specialized Hospital between July 1, 2019, and June 30, 2022

To obtain the largest sample size possible, we used online open-epi version 3 software with a case-to-control ratio of 1:4, a power of 80%, and a confidence interval of 95%. Variables from various published journals (nutritional status: 12.5% [24], season of admission: 20.06% [21], antibiotic changes considered: 26.6% [33], and time of presentation: 28.13% [27]) were used as exposed controls in patients with acute bacterial meningitis who were alive at discharge. To include the maximum number of study participants, all 355 eligible (71 cases and 284 controls) medical charts of children diagnosed with and treated for acute bacterial meningitis were included in a case-to-control ratio of 1:4. Using the inclusion criteria, the sampling frame was prepared for cases and controls from a serial list of pediatric medical record numbers, which were taken from integrated admission and discharge logbooks in the NICU and pediatrics ICU. Then, 355 pediatric medical record numbers were taken consecutively to extract charts from the hospital archive room. The medical recording numbers of charts were selected using the procedure in Fig. 1.

### Study variables

#### Dependent variables

Mortality due to acute bacterial meningitis.

#### Independent variables

##### Sociodemographic factors

Family size, residence, age, sex.

##### Clinical factors

duration of illness before presentation, loss of consciousness, increased intracranial pressure, comorbidity, coma, seizures, nutrition status, breastfeeding status, and immunization status.

##### Bacterial pathogen factors

cerebrospinal fluid glucose, cerebrospinal fluid protein, bacterial species.

##### Treatment-related

Antibiotic regimen, corticosteroid administration, season of admission, antibiotic regimen change.

### Data collection procedure and data quality management

The data abstraction tool was developed after a thorough literature review of published studies on bacterial meningitis in pediatrics [10, 16, 17, 21–24, 26, 30, 34]. Two BSc nursing professionals and one MPH supervisor were assigned to the data collection and supervision process. Before the actual data collection started, one day of training was given to data collectors and supervisors on how to collect and record data appropriately. Charts of pediatric patients admitted to the pediatric ward due to acute bacterial meningitis were used to collect the data. The data abstraction tool was pretested with 5% of the sample size, and slight modifications were made. Based on registration numbers, all charts were identified by the study team and provided for data collectors. Then, from all 355 (71 cases and 284 controls) charts, relevant patient data (i.e., sociodemographic, clinical features at presentation, pathogen-related factors, treatment, and final discharge outcome (died or discharged alive)) were extracted by data collectors.

### Data analysis procedures

The collected data were checked for completeness and consistency and cleaned, then entered into Epi-Data version 4.6, and then transferred to SPSS version 25 for analysis. Descriptive statistical analysis, such as frequency and proportion, was used to describe the study participants. Binary logistic regression analysis was carried out, and the candidate variables were selected at a *p* value of 0.25. Multiple logistic regression was performed, and independent determinants of acute bacterial meningitis mortality were identified at a.

*p* value of 0.05 along with a 95% CI. Meanwhile, the model fitness of the study was assessed using the Hosmer and Lemshow model fitness test (X2 = 7.29, *P* value = 0.51). The results were finally presented using tables and figures.

### Operational definitions

**Bacterial meningitis** was defined according to the physician’s clinical diagnosis, including either laboratory-confirmed or probable cases and if no changes (improvement) in treatment were considered until discharge or death due to other causes of meningitis, such as tuberculosis [15].

**Acute bacterial meningitis** is defined in this study as an abrupt onset with progression over hours of clinical symptoms (fever, headache, seizure, vomiting and impaired consciousness) within less than 4 weeks of bacterial infection [35].

#### Acute bacterial meningitis mortality

pediatric death due to the presumptive diagnosis of acute bacterial meningitis infection [36].

**Antibiotic regime changes** were defined as a change in empirical antibiotics within 2–3 days in cases where the patient was not improving with initial empiric antibiotics [22].

**Increased intracranial pressure** defined according to the physician’s clinical diagnosis, such as focal paralysis in any of the limbs, vomiting, headache in older children, bulged fontanel in < 2 years, unequal pupils, irregular breathing, bradycardia and low blood pressure on examination [15].

**Young infants** were defined in this study as < 2 months of age, and **older infants and children** as 2 months to 14 years of age, based on the treatment-protocol difference [22].

**Malnourished** considered children who were moderately malnourished (z score between negative 3 and 2) or severely malnourished (Z score less than negative 3) [30].

Consciousness was assessed using the Glasgow Coma Scale (GCS) score for children > 2 years old and the Pediatric Glasgow Coma Scale (PGCS) in children < 2 years old (**eye opening**: 4 = spontaneous, 3 = to sound, 2 = to pain, 1 = none: **verbal response**: 5 = age-appropriate vocalization, smile, or orientation to sound, interacts (coos, babbles), follows objects, 4 = Cries4 = cries, irritable, 3 = cries to pain, 2 = moans to pain, **1 = none: motor response**: 6 = spontaneous movements (obeys verbal command) 5 = withdraws to touch (localizes pain), 4 = withdraws to pain, 3 = abnormal flexion to pain (decorticate posture), 2 = abnormal extension to pain (decerebrate posture), 1 = none), which has a score of 3 to 15. **Full consciousness** refers to a score of 15; a score of 9–14 was considered **impaired consciousness**, and patients with a score of 8 or less were classified as **comatose** [22, 37].

## Results

A total of 423 pediatric charts (73 cases and 350 controls) for children admitted to the pediatric ward at WSUCSH from July 1, 2019, to June 30, 2022, with a diagnosis of bacterial meningitis were reviewed during the study period. Approximately 68 patient charts were excluded from the charts reviewed. Fifteen (two cases and 13 controls) patients’ charts were excluded from the study for the following reasons: 8 (one case was diagnosed with tuberculosis meningitis, and seven controls’ diagnoses changed after lumbar puncture and CSF analysis). Three (1 case and 2 control charts with incomplete clinical information) and four [4] control diagnoses were changed by the treating physician within 48 h of the initiation of treatment for an alternative diagnosis. Eleven patients were referred to other health institutions, and 18 were discharged against medical advice. Approximately 24 (5%) of the charts used for the pretest were also excluded from the study. Therefore, data collection and analysis were performed for 355 (71 cases and 284 controls) patient charts.

### Sociodemographic characteristics of the children

Among 71 cases and 284 controls enrolled in the study, 45 (63.4%) cases and 162 (59.5%) controls were males. Young infants made up 6 (8.5%) cases and 38 (13.1%) controls, while more than half of the cases, 37 (52.1%) and 102 (35.9%) controls, were children between the ages of 2 months and 5 years. Between the ages of 5 and 10 years, there were 23 (32.4%) cases and 101 (35.6%) controls, while between the ages of 10 and 14 years, there were 5 (7%) cases and 43 (15.1%) controls. The average family size was approximately 5 among study subjects, with a minimum of 3 and a maximum of 11 for both cases and controls. Among the study subjects, more than half (49( 69%) of the cases and 112 (39.3%) of the controls were from large families (more than or equal to 6). More than half (39(54.9%) of the cases and the majority (235, 82.7%) of the controls were admitted between September and May. More than three-fourths of 58 (81.7%) cases and nearly three-fourths of 205 (72.2%) controls were from a rural area (Table 1).

**Table 1:** Demographic characteristics of pediatric patients with acute bacterial meningitis admitted to Wolaita Sodo University Comprehensive Specialized Hospital between July 1, 2019, and June 30, 2022

Variables	Category	Controls: n (%)	Cases: n (%)	*P* value
**sex of the child**	Male	169 (59.5)	45 (63.4)	0.55
	Female	115(40.5)	26(36.6)	
**Age of the child**	< 2 months	38(13.1)	6(8.5)	0.049
	2 months to 5 years	102(35.9)	37(52.1)	0.026
	5–10 years	101(35.6)	23(32.4)	0.201
	10–14 years	43(15.1)	5(7.0)	
**Area of residence**	Rural	205(72.2)	58(81.7)	0.102
	Urban	79(28.2)	13(18.3)	
**Season of admission**	Summer	49(17.3)	32(45.1)	< 0.001
	Winter	235(82.7)	39(54.9)	
**Family size**	less or equal to 5	172(60.7)	22(31.0)	< 0.001
	Greater than or equal to 6	112(39.3)	49(69.0)	

### Clinical factors

In more than half of the cases, 41 (57.7%) and 93 (32.7%) controls presented after the illness had been present for more than three days. Additionally, a history of loss of consciousness was reported by 68 (94.8%) cases and 135 (47.5%) controls, while a history of seizure was reported by 68 (95.5%) cases and 230 (81.0%) controls. All 71 (100%) cases and most (274(96.6%) controls presented with a history of fever. Forty-two cases (59.2%) and 34 controls (12.0%) showed elevated ICP at presentation.

Approximately two-thirds, 47 (66.2%) of cases and 220 (77.5%) controls, had been exclusively fed breast milk. More than half, 38 (53.5%) cases and 88 (31.0%) controls, were unvaccinated for their age. Nearly half of the 32 cases (45.1%) and one in ten, 31 (10.9%) controls were malnourished. Thirty-two (45.1%) cases and 120 (42.3%) controls had impaired consciousness. Half, 36 (50.7%) cases and 22 (7.7%) controls, were comatose in their presentations.

Pneumonia was present in 25 (13 cases and 12 controls), while malaria was present in 4 controls. Four patients presented with hypovolemic shock (3 cases and 1 control), five patients presented with CHF (3 cases and 2 controls), and more than one comorbidity was present in 16 (22.5%) cases and 13 (4.6%) controls (Table 2).

**Table 2:** Clinical presentations of pediatric patients with acute bacterial meningitis admitted to Wolaita Sodo University Comprehensive Specialized Hospital between July 1, 2019, and June 30, 2022

Variables	Category	Controls: n (%)	Cases: n (%)	*P* value
Loss of consciousness	No	149 (52.5)	3 (4.2)	< 0.000
	Yes	135 (47.5)	68 (95.8)	
Abnormal body movement	No	54 (19)	3 (4.2)	0.002
	Yes	230 (81)	68 (95.8)	
Increased ICP	No	250 (88)	29 (40.8)	< 0.000
	Yes	34 (12.0)	42 (59.2)	
Exclusively breastfeed	No	64 (22.5)	24 (33.8)	0.049
	Yes	220 (77.5)	47 (66.2)	
Vaccinated for his/her age	No	88 (31.0)	38 (53.5)	< 0.001
	Yes	196 (69.0)	33 (46.5)	
Nutritional status	Malnourished	31 (10.9)	32 (45.1)	< 0.001
	Normal	253 (89.1)	39 (54.9)	
Glasgow coma scale	Conscious	142 (50.0)	3 (4.2)	
	Impaired	120 (42.3)	32 (45.1)	< 0.001
	Comatose	22 (7.7)	36 (50.7)	< 0.001
Comorbidity categorical	None	232 (81.7)	38 (53.5)	
	only one comorbidity	39 (13.7)	17 (23.9)	< 0.001
	More than 1	13 (4.6)	16 (22.5)	0.028
Body temperature	Afebrile	158 (55.6)	32 (45.1)	0.110
	Febrile(> 38℃)	126 (44.4)	39 (54.9)	
Duration categorical	<= 3 days	191 (67.3)	30 (42.3)	< 0.000
	> 3 days	93 (32.7)	41 (57.7)	
Types of Comorbidities	Pneumonia	25 (8.8)	13 (18.3)	< 0.000
	Malaria	4 (1.4)	0	0.999
	AGE	5 (1.8)	0	0.999
	DM	2 (0.7)	0	0.400
	Hypovolemic shock	1 (0.4)	3 (4.2)	0.158
	CHF	2 (0.7)	3 (4.2)	

### Treatment-related factors

Forty-two (59.2%) cases and 247 (76.9%) controls received ceftriaxone as their first antibiotic, whereas 28.2% of cases and 37 (11.5%) controls received ceftriaxone with vancomycin. Ampicillin and gentamicin were used to treat 37 (11.5%) controls and 9 (12.7%) cases, respectively. Only 72 patients had their first antibiotic modified 30 (42.3%) cases and 42 (13.1%) controls). Ceftazidime and vancomycin were the most frequently changed antibiotics [19 (63.3%) cases and 28 (63.6%) controls]. Dexamethasone was not given to more than 75% of the patients, 62 (87.3%) cases and 265 (82.6%) controls (Table 3).

**Table 3:** Antibiotics treatment pattern of pediatric patients with acute bacterial meningitis admitted to Wolaita Sodo University Comprehensive Specialized Hospital between July 1, 2019, to June 30, 2022

Variables	Category	Controls :n(%)	Cases: n(%)	*p* value	
Antibiotics regimen	Ceftriaxone	218(76.8%)	42(59.2%)	0.002	
	ceftriaxone with vancomycin	32(11.3%)	20(28.2%)	0.001	
	ampicillin with gentamycin	34(12%)	9(12.7%)	0.068	
Antibiotics change	No	245(87.7%)	41(57.7%)	< 0.000	
	Yes	39(12.3%)	30(42.3%)		
The type of antibiotics changed	Vancomycin	7(18.9%)	6(20.0%)		
	ceftazidime with vancomycin	23(62.2%)	19(63.3%)	0.998	
	Cefotaxime	1(2.7%)	1(3.3%)	0.76	
	ceftriaxone with Vanco	6(16.2%)	4(13.3%)		
Dexamethasone	No	235(82.7%)	62(87.3%)		0.351
	Yes	49(17.3%)	9(12.7%)		

### Pathogen-related factors

The most frequently detected bacterial pathogen among 355 pediatric patients (71 cases and 284 controls) studied was S. pneumoniae 35 (15.8%) [in 5 (55.6%) cases and 30 (14.2%) controls compared with other bacterial etiologies], followed by N. meningitidis 31 (14%) [2 (22.2%) in cases and 29 (13.7%) controls]. Group B streptococcus (GBS) (5.23%) and H. influenza (12.44%) in controls. Only one of the cases and 138 (62.4%) of the controls with CSF analysis revealed a bacterial pathogen, and only one of the cases and 58 (27.2%) of the controls with CSF glucose were less than 70 mg, while the rest were greater than 70 mg. Slightly more than 6 (66.7%) of the cases and 139 (65.6%) of the controls had CSF protein levels greater than or equal to 100 mg, while 85 (40.1%) of the controls had cell counts below 50, 56 (26.4%) below 50, and 1 (11.1%) between 50 and 100 mg. In terms of cell count, 8 (88.9%) cases, 71 (33.5%) controls, and 79 (35.7%) cases all had more than 100 cells (Table 4).

**Table 4:** Pathogen-related factors of pediatric patients with acute bacterial meningitis admitted to Wolaita Sodo University Comprehensive Specialized Hospital between July 1, 2019, and June 30, 2022

Variables	Category	Control:n(%)	Cases: n(%)	*p* value
Was Lumbar puncture done	No	109 (38.4)	62 (87.3)	< 0.000
	Yes	175 (61.6)	9 (12.7)	
Types of bacteria detected, n = 9 for cases n = 175 for controls	S.pneumonia	18 (10.3)	5 (55.6)	0.001
	N.meningitidis	25 (14.3)	2 (22.2)	0.130
	Group B Streptococcus	4 (2.3)	0 (0.0)	0.999
	H. influenza	10 (5.7)	0 (0.0)	0.999
	not detected	118 (67.4)	2 (22.2)	
CSF Glucose	< 70	52 (29.9)	1(11.1)	0.226
	> 70	122 (70.1)	8 (88.9)	
CSF Protein	less than 100	72 (41.1)	3 (33.3)	0.642
	>= 100	103 (58.9)	6 (66.7)	
CSF cell count	< 50	74 (42.3)	0	
	50–100	48 (27.4)	1 (11.1)	0.997
	> 100	53 (30.3)	8 (88.9)	0.067

### Determinants of acute bacterial meningitis mortality

In multivariate analysis, the season of admission, the pediatric age, the size of the family, delay in presentation, clinical features such as loss of consciousness, seizures, and increased intracranial pressure, as well as comorbidity, malnutrition, and initial antibiotic change, were found to be determinants of an increased risk of acute bacterial mortality.

The odds of acute bacterial meningitis mortality among pediatric patients aged between 2 months and 5 years was 3 times higher than that among pediatric patients aged between 10 and 14 years (AOR = 2.95, 95% CI = 1.05, 8.33).

Pediatric patients who were admitted in the winter had 73% lower odds of dying than those who were admitted in the summer (AOR = 0.274, 95% CI = 0.16–0.50).

The odds of mortality of acute bacterial meningitis among pediatric patients who presented after three days of symptom development were almost two [2] times those who sought health care centers earlier (AOR = 2.19, 95% CI = 1.12,4.28).

The mortality of acute bacterial meningitis had 57% lower odds among fully vaccinated pediatrics than among incomplete or unvaccinated pediatrics (AOR = 0.47, 95% CI= (0.23–0.95).

Additionally, the odds of developing meningitis mortality among patients who had six or more family members had a threefold increased risk of death in comparison to the control groups (AOR = 3.10, 95% CI = 1.73–5.55).

The odds of developing meningitis mortality among patients who experienced a loss of consciousness (AOR = 16.85, 95% CI = 4.70-60.39), abnormal body movements (seizures) (AOR = 6.51, 95% CI = 1.82–23.35), and increased intracranial pressure (AOR = 3.63, 95% CI = 1.78–7.38) were 13, 5, and 2 times the risk of passing away compared to patients who did not experience these symptoms, respectively.

Patients who were undernourished had odds of dying that were almost three times higher than those who were well-fed (AOR = 4.04, 95% CI = 1.83–8.89).

Similarly, patients with more than one comorbidity had almost twice the increased risk of dying (AOR = 1.84, 95% CI = 1.13, 2.99).

Pediatric patients whose initial antibiotic therapy was modified had approximately 11 times the risk of dying than those who had finished initial antibiotic treatment (AOR = 10.81, 95% CI = 2.10, 5.71) (Table 5).

**Table 5:** Determinants of acute bacterial meningitis mortality among pediatric patients admitted to Wolaita Sodo University Comprehensive Specialized Hospital between July 1, 2019, and June 30, 2022

Variables	Category	Controls n(%)	Cases n(%)	COR(95%CI)	AOR(95%CI)	*p* value
Age of child	2mon-5yrs*	102(35.9)	37(52.1)	2.30(0.90,5.89)	2.95(1.05,8.33))	0.041
	10–14 yrs	43(15.1)	5(7.0)			
Area of residence	Urban	79(27.8)	13(18.3)	0.58(0.30,1.12)		
	Rural	205(72.2)	58(81.7)		NA	
season of admission	Winter*	238(82.7)	39(54.9)	0.254(0.15,0.45)	0.278(0.15,0.50)	< 0.001
	Summer	49(17.3)	32(45.1)		NA	
family size	>=6 *	112(39.4)	49(69)	3.42(1.96,5.97)	3.10(1.73,5.55)	< 0.001
	<= 5	172(60.7)	22(31.0)		NA	
Duration before presentation	<= 3 days	191 (67.3)	30 (42.3)			
	> 3 days	93 (32.7)	41 (57.7)	2.81(1.65,4.78)	2.19(1.12,4.28)	< 0.001
Loss of consciousness	Yes*	135(47.5)	68(95.8)	25.02(7.69,8138)	16.85(4.7,60.39)	< 0.001
	No	149 (52.5)	3 (4.2)		NA	
Seizure	Yes*	230(81.0)	68(95.8)	5.32(1.61,17.56)	6.51(1.82,23.35)	0.004
	No	54 (19)	3 (4.2)		NA	
increased ICP	Yes*	29(12.8)	42(59.2)	10.65(5.88,19.78)	3.63 (1.78,7.38)	< 0.001
	No	250 (88)	29 (40.8)		NA	
Nutritional status	Malnourished*	31(10.9)	32(45.1)	6.70(3.68,12.18)	4.04(1.83,8.89)	0.001
	Normal	253 (89.1)	39 (54.9)			
Vaccination	Vaccinated for age*	196 (69.0)	33 (46.5)	0.39(0.23,0.66)	0.47(0.23,0.95)	0.034
	Not vaccinated	88 (31.0)	38 (53.5)		NA	
Comorbidity	> 1 disease*	13(4.6)	16(22.5)	2.27(1.87,3.96)	1.84(1.13,2.30)	0.0.015
	None	232 (81.7)	38 (53.5)		NA	
	only one comorbidity	39 (13.7)	17 (23.9)		NA	
Treatment	Ceftriaxone with Vanco *	32(11.3)	20(28.2)	2.22(0.90,5.52)		
	Ceftriaxone	218(76.8)	42(59.2)			
Antibiotics change	Yes*	35(12.3)	30(42.3)	5.21(2.89,9.38)	10.81(2.01,5.71)	0.004
	No	249(87.7)	41(57.7)			
Pathogen	S.pneumonia *	18(10.3)	7(9.9)	11.33(2.10,61.22)		
	N.meningitidis *	25(14.3)	2(2.8)	4.69(0.63,34.67)		

* Variables of p value < 0.05 in bivariate analysis, NA = not applicable because P value > 0.25 in bivariate analysis

## Discussion

According to this facility-based unmatched case‒control study, the age of the child, the season of pediatric admission, family size > = 6, initial antibiotic change, clinical features at presentation such as loss of consciousness, abnormal body movement, increased intracranial pressure, malnutrition, vaccination, and the presence of more than one comorbidity were the major determinants of mortality in this study setup.

Based on the findings of this study, the odds of mortality among patients between the ages of 2 months and 5 years were higher than those among patients between 10 and 14 years. This finding coincides with a study conducted in Bangladesh [30]. This is closely related to the age group’s inadequate immunity and high burden of bacterial infections among these ages.

Children from large families ( > = 6) had a greater rate of acute bacterial meningitis mortality than children from smaller families ( < = 5). This is consistent with research conducted in Madagascar [27]. Increased household population density has been regarded as a subtle indicator of overcrowding and lack of hygiene, which have increased the risk of bacterial meningitis.

The results showed that pediatric patients admitted during the winter had a lower rate of death than patients hospitalized throughout the summer season. This study was consistent with another study carried out in Ethiopia [21]. The rainy season exacerbates the weak infrastructure of the setting and limited funding due to low cash crop yields during the rainy season, resulting in late visits to healthcare centers throughout the summer season.

Pediatric patients who are fully vaccinated according to the Ethiopian programme immunization schedule have a lower chance of death than their counterparts. This finding is consistent with a study performed in Afghanistan [28]. It is known that the most common virulent causes of bacterial meningitis are vaccine preventable, and vaccinated pediatrics are more immune than those who are not vaccinated.

In this study, mortality among children who presented 3 days after the onset of symptoms was higher than among those who visited earlier. This finding agrees with other studies conducted in Finland, Angola, Latin America [26], Madagascar [27], and Ethiopia [24]. It is well recognized that late presentation after advanced clinical features, in conjunction with an inadequate critical care unit in a health service center, makes curative therapy of patients challenging, resulting in higher mortality.

Before or at the time of hospital admission, individuals with severe illness symptoms such as loss of consciousness, abnormal body movements (seizures), and increased intracranial pressure were observed to have a greater acute bacterial meningitis death rate than patients without these clinical signs and symptoms. Various studies have reported comparable results [16, 23, 24, 28, 38]. The possible reason for this higher mortality might be related to late health-seeking behavior, poor infrastructure and weak referral linkage of primary health institutions, and limited advanced health care for these patients.

This study also showed that patients with malnutrition had greater mortality rates than those who were receiving adequate nutrition. This finding is consistent with similar findings made in Bangladesh [30] and Ethiopia [20, 24]. Thus, the mortality of bacterial meningitis in developing countries is higher than that in developed countries [36]. This may be related to the fact that children who have malnutrition are believed to be immunosuppressed, which can aggravate and complicate infection during treatment. This could also be associated with a lack of advanced care specifically for patients admitted with undernourishment and meningitis.

Patients in this study who had more than one underlying illness died of acute bacterial meningitis at a greater rate than those who had only one or no comorbidities. The study findings are consistent with those of other investigations [16]. Comorbidity weakens the patient’s immune system and provides conditions favorable to the development and fatality of acute bacterial meningitis.

Changes in initial antibiotics were strongly correlated with death from acute bacterial meningitis compared with children who had finished their course of therapy with initial antibiotics. This study conforms to prior research conducted in Ethiopia [16, 23]. This is linked to delayed drug switches and a poor referral system from primary care facilities after the start of antibiotics.

Furthermore, the fragile health system, a health infrastructure lacking basic facilities such as microbiological diagnosis and intensive care for critically ill patients, the weak referral link between primary care and tertiary centers, and the lack of health education were contributors to acute bacterial mortality in pediatrics [28].

### Limitations of the study

The study was founded on documented data (chart review) and might not have included all the variables that were not listed in the patient files, and those discharged against medical advice are totally missed. Errors may also have occurred because of the use of clinical diagnosis rather than laboratory-based diagnostics, which may have been less accurate. The retrospective approach of the study might also be a drawback. In some situations, mortality may occur after being discharged alive.

## Conclusion

In the study setup, the age of the child, the season of pediatric admission, family size, and clinical features such as loss of consciousness, abnormal body movement, increased intracranial pressure, malnutrition, the presence of more than comorbidity, and the change in initial antibiotics were predictors of mortality due to acute bacterial meningitis.

### Electronic supplementary material

Below is the link to the electronic supplementary material.

Supplementary Material 1

## Data Availability

The datasets used and/or analyzed during this study are available from the corresponding author upon reasonable request.

## Abbreviations

AOR: Adjusted odds ratio
CI: Confidence interval
CSF: Cerebrospinal fluid, ENT, nose throat
Hib: Haemophilus influenza type b
LatAm: Latin America
MACV: Meningococcal A vaccine
NICU: Neonatal intensive care unit
PICU: Pediatric intensive care unit
WHO: World Health Organization
WSUCSH: Wolaita Sodo University Comprehensive Specialized Hospital
