# Supplementary material for: Determinants of mortality among pediatric patients admitted to Wolaita Sodo University Comprehensive Specialized Hospital with acute bacterial meningitis, Southern Ethiopia: an unmatched case–control study
Source: BMC Pediatr. 2023 Dec 4;23:610. doi: 10.1186/s12887-023-04410-6 (PMC10694971; doi:10.1186/s12887-023-04410-6)
Supplement: Supplementary file 1 — Supplementary Material 1 [file 12887_2023_4410_MOESM1_ESM.docx]

Checklist of items

|  | Item No. | Recommendation | Page  No. |  |
| --- | --- | --- | --- | --- |
| **Title and abstract** | 1 | (*a*) acute bacterial meningitis, an unmatched case control study | 1 |  |
|  |  | (*b*) Provide in the abstract an informative and balanced summary of what was done and what was found | 2 |  |
| Introduction | | | |  |
| Background/rationale | 2 | Explain the scientific background and rationale for the investigation being reported | 3,4 |  |
| Objectives | 3 | To identify determinants of mortalty from acute bacterial meningitis in children | 3 |  |
| Methods | | | |  |
| Study design | 4 | Facility based an unmatched case control study | 5 |  |
| Setting | 5 | This study was conducted at Wolaita Sodo University Comprehensive Specialized Hospital from July 01, 2019, to June 30, 2022. | 5 |  |
| Participants | 6 | The study included all pediatric patients who were hospitalized with a presumptive diagnosis of acute bacterial meningitis between July 1, 2019, and June 30, 2022. Deaths confirmed by a physician were considered as cases, while those recovered or alive at discharge were considered controls. | 5 |  |
| Variables | 7 | Acute bacterial meningitis mortality:pediatric death due to presumptive diagnosis of acute bacterial meningitis infection, family size, age, sex, residience, The duration of illness before presentation, Loss of consciousness, increased ICP, Comorbidity, Coma ,Seizures, Nutritional status, Breastfeeding, Immunization status, Cerebro-spinal fluid glucose , Cerebro-spinal fluid protein, Bacterial species, Antibiotics regimen, Corticosteroid administration, Season of admission, Antibiotics regimen change | 6 |  |
| Data sources/ measurement | 8* | Charts of pediatric patients admitted to the pediatric ward due to acute bacterial meningitis were used to collect the data. Based on registration numbers, all charts reviewed and charts that met the inclusion criteria identified from the medical archive room, then data extraction was made from 355(71 cases and 284 controls) charts by data collectors, and  relevant patient data (i.e., socio-demographic, clinical features at presentations, pathogen-related factors, treatment, and final discharge outcome (died or discharged alive)) were extracted. | *7* |  |
| Bias | 9 | All case and control charts were selected based on serial number by principal investigator and provided for data collectors |  |  |
| Study size | 10 | The maximum sample size was determined using online open-epi version 3 software after input of case to control ratio 1:4, power 80%, and confidence interval 95%. About 12.5%, 20.06%, 26.6%, and 28.13% of controls were expected to be exposed for acute bacterial meningitis, respectively. | 6 |  |

Continued on next page

| Quantitative variables | 11 |  |  |  |
| --- | --- | --- | --- | --- |
| Statistical methods | 12 | (*a*) Data entered to Epi-Data version 4.6, transferred to SPSS version 25 for analysis. Binary logistic regression analysis was carried out then Multiple logistic regression was done and independent determinants of acute bacterial meningitis mortality were identified at a p-value of 0.05 along with a 95% CI. Meanwhile, the model fitness of the study was assessed using the Hosmer and Lemshow model fitness test. | 7 |  |
| Results | | | | |
| Participants | 13* | (a) A total of 423 pediatric charts (73 cases and 350 controls) for children admitted to the pediatric ward at WSUCSH from July 1, 2019, to June 30, 2022, with a diagnosis of bacterial meningitis were reviewed during the study period. |  | 8 |
|  |  | (b) About 68 patient charts were excluded from the total number of charts reviewed due to incomplete chart, Tuberculosis meningitis, alternative diagnosis, referral and discharge against medical adivice, and antibiotic regimen change |  | 8 |
|  |  | (c) figure 1 |  |  |
| Descriptive data | 14* | (a) Among 355(71 cases and 284 controls) charts enrolled in the study; 45 (63.4%) cases and 162 (59.5%) of the controls were males. |  | 8 |
|  |  | *A*mong 355 participants 71 were died and 284 were discharged alive |  |  |
| Main results | 16 | The season of admission, the pediatric age, the size of the family, delay in presentation, clinical features such as loss of consciousness, seizures, and increased intracranial pressure, as well as comorbidity, malnutrition, and initial antibiotic change, were found to be determinants of an increased risk of acute bacterial mortality. |  | 10-11 |

Continued on next page

| Discussion | | | | |
| --- | --- | --- | --- | --- |
| Key results | 18 | Discussion |  | 11 |
| Limitations | 19 | limitations of the study |  | 14 |
| Interpretation | 20 | According to this facility based an unmatched case-control study, the age of the child, the season of pediatric admission, family size, initial antibiotic change, clinical features at presentations like loss of consciousness, abnormal body movement, increased intracranial pressure, malnutrition, vaccination and the presence of more than one comorbidity were the major determinants of mortality in this study setup. |  | 11-13 |
| Other information | |  | | |
| Funding | 22 | Not available |  | 15 |
